# Systematic Review Evaluating the Influence of Veneering Design on the Clinical Outcome of All‐Ceramic Implant‐Supported 3–4 Units and Full Arch Fixed Dental Prostheses

**DOI:** 10.1111/jerd.70177

**Published:** 2026-05-09

**Authors:** Shaza Bishti, Stefan Wolfart, Marcel Zwahlen, Lukas Waltenberger

**Affiliations:** ^1^ Department of Prosthodontics and Biomaterials, Centre for Implantology Uniklinik RWTH Aachen Aachen Germany; ^2^ Institute of Social and Preventive Medicine University of Bern Bern Switzerland; ^3^ Bern University of Applied Sciences School of Health Professionals Bern Switzerland

## Abstract

**Objective:**

To systematically assess survival, failure, and complication rates of all‐ceramic implant‐supported monolithic and micro‐veneered FDPs (i‐FDPs) in partially/completely edentulous patients compared to veneered restorations.

**Materials and Methods:**

An electronic search identified prospective clinical studies on all‐ceramic i‐FDPs with at least one inter‐implant pontic and a minimal follow‐up of 1 year, with no restrictions in language and publication date.

**Results:**

Only studies on posterior 3–4‐unit and full‐arch i‐FDPs were identified. Twelve studies assessing monolithic, fully veneered, and micro‐veneered restorations were included. Regardless of veneering design, the meta‐analysis indicated a 3‐year success rate of 89.36% for 3–4‐unit and 75.56% for full‐arch i‐FDPs, and an estimated 3‐year survival rate of 98.64% and 96.45%, respectively. Analyzing veneering design influence was feasible only for 3–4‐unit i‐FDPs. Monolithic and micro‐veneered 3–4‐unit restorations showed significantly lower chipping incidence than fully veneered ones (*p* < 0.0001). The estimated annual chipping rate was 2.1% for 3–4‐unit i‐FDPs and 6.9% for full‐arch restorations.

**Conclusion:**

Monolithic/micro‐veneered designs are preferred for short‐span i‐FDPs to minimize chipping, whereas full‐arch all‐ceramic restorations require caution due to limited evidence and high complication rates. Current findings are based solely on high‐strength 3Y‐TZP zirconia (> 1000 MPa). No conclusions can be drawn for multilayer zirconia or other ceramics.

## 1 Introduction

Significant advances in the field of dental ceramics as well as the expanding demand for metal‐free restorations have contributed to the increasing popularity and reliability of all‐ceramic restorations [1, 2]. Moreover, innovations in CAD/CAM technologies and the development of improved restorative materials have further expanded their clinical applications to include long‐span fixed dental prostheses and even full‐arch reconstructions [3, 4, 5, 6]. Zirconia has been instrumental in this progress due to its high mechanical strength, esthetics, and compatibility with digital fabrication, improving efficiency and reducing costs compared to conventional techniques [7, 8, 9, 10].

For implant‐supported single crowns, a range of ceramics is now available, including lithium silicate‐reinforced glass ceramics, zirconia with varying yttria content, and polymer‐infiltrated ceramic networks [7, 11, 12]. In contrast, multi‐unit implant‐supported FDPs are typically limited to metal ceramics and zirconia due to strength demands and regulatory approval [13, 14]. Long‐term evidence still favors metal‐ceramic restorations, with a 5‐year survival rate of 98.7% versus 93% for zirconia [15], although recent advances in full‐ceramic systems are narrowing this gap.

Following the success of tooth‐supported ceramic FDPs, zirconia has been widely adopted for implant‐supported single crowns and FDPs, owing to its proven biocompatibility and mechanical durability, even in posterior load‐bearing zones [1, 2, 16, 17, 18, 19]. However, due to their high opacity, early zirconia ceramics of the first generation (3Y‐TZP with 0.25% aluminium oxide) could not meet esthetic expectations and needed to be veneered with a layer of feldspathic ceramic of low flexural strength. These veneering systems are prone to delamination failures, chipping, and fractures of the veneering [20]. A high annual chipping rate of 13.9% for implant‐supported FDPs has been reported [15, 21]. Moreover, a clinical study investigating the outcome of implant‐supported restorations in posterior areas reported high fracture rates of the veneering ceramic, with a survival rate of only 43.2% after an observation period of 13 years [22].

With the availability of tooth‐colored second‐generation zirconia (3Y‐TZP with 0.05% alumina oxide), monolithic structures also known as solid, full‐contour restorations were introduced to effectively reduce the risk of chipping [23]. These exhibit high flexural strength, reduce the unwanted complication of chipping, and require less laboratory time since the need of sophisticated manual veneering is eliminated [23]. However, the main disadvantage of second‐generation monolithic materials was their high opacity, which led to low esthetic performance, and their use was therefore restricted to the posterior areas, where no esthetic demand was required [23]. In order to improve the esthetic characteristics, contemporary translucent zirconia ceramics with enhanced optical properties were developed. Here, a higher concentration of yttrium is used, resulting in partially stabilized zirconia (4Y‐TZP, 5Y‐TZP or 6‐Y‐TZP). Such changes in the chemical composition result in an increased amount of monoclinic zirconia at room temperature, which led to improved esthetic characteristics by increasing translucency but resulted in reduced mechanical strength as low as 600 MPa [23, 24]. Therefore, the clinical indications of 4Y‐TZP, 5Y‐TZP, and 6‐TZP zirconia are consecutively more restricted in comparison to 3Y‐TZP.

A reduced form of classical buccal veneering, also known as micro‐veneering, is an alternative to enhance esthetics of monolithic, tooth‐colored restorations. Here, a reduced veneering layer with a maximum thickness of 0.5 mm is applied strictly to the non‐functional areas only. The incisal edges and/or occlusal surfaces remain completely monolithic to prevent the incidence of veneer fracture [25]. Data in the literature prospectively evaluating monolithic and/or micro‐veneered implant‐supported FDPs are rare. A recent systematic review, which included retrospective studies, recorded equal survival rates for monolithic or micro‐veneered implant‐supported FDPs in the posterior region after an observation period of up to 3 years [26]. On the other hand, little differences in the survival rate of veneered zirconia‐ceramic FDPs compared to monolithic restorations were reported [26]. Monolithic restorations showed lower ceramic fracture and chipping rates than veneered restorations in general, regardless of which material was used.

The need for both mechanically high strength and esthetically acceptable restorations led to the development of modern ceramics. More recently, graded zirconia blocks, also known as multilayer materials, have been developed and aim to mimic the dental structure as much as possible. These materials blend different types of zirconia along their structure, with a stronger structure at the base (3Y or 4Y‐TZP), which ensures high mechanical strength, and a more translucent zirconia at the surface (4Y or 5Y‐TZP), which guarantees acceptable esthetics [27, 28]. In vitro studies investigating the mechanical behavior and optical properties of graded zirconia reported higher mechanical properties of monolithic single crowns made from graded zirconia in comparison to lithium disilicate crowns, whereas lithium disilicate reported high optical properties and better translucency than the graded zirconia ones [27, 29, 30]. However, high‐quality clinical data of strength‐graded zirconia on multi‐unit implant‐supported FDPs is scarce yet highly demanded to provide guidelines for everyday clinical practice.

To the best knowledge of the authors, information regarding the success, survival, complication, and failure rates of all‐ceramic multi‐unit i‐FDPs with special respect to their veneering design is lacking. The presence of an inter‐implant pontic introduces additional biomechanical challenges, as it alters the load distribution and increases flexural and tensile stresses within the restoration and supporting implants. These mechanical demands are particularly critical for all‐ceramic materials, which, despite their advancements in strength and fracture resistance, remain more susceptible to tensile forces compared to metal‐based alternatives. Understanding the long‐term survival, failure patterns, and complication rates of such restorations is essential for optimizing material selection and prosthetic design to ensure predictable clinical outcomes. Therefore, the aim of the present systematic review was to answer the following question: “In partially or completely edentulous patients, do monolithic all‐ceramic i‐FDPs exhibit differences in survival, failure, and complication rates when compared to veneered all‐ceramic i‐FDPs?”

## 2 Materials and Methods

### 2.1 Protocol Development

This systematic review was conducted in accordance with the guidelines of the Preferred Reporting Items for Systematic Review and Meta‐analysis (PRISMA) statement. The review protocol was prospectively registered with PROSPERO (International prospective register of systematic reviews) (registration number: CRD42022361524; Link https://www.crd.york.ac.uk/PROSPERO/view/CRD42022361524).

The following PICOS‐criteria were defined:

- P: Population: Partially or completely edentulous patients restored with implant‐supported reconstructions with at least one inter‐implant pontic.
- I: Intervention: Dental implants restored with monolithic or micro‐veneered all‐ceramic fixed dental prosthesis.
- C: Comparison: Dental implants restored with veneered all‐ceramic fixed dental prosthesis.
- O: Outcome: Survival, success, failure, and complication rates (biological/technical).
- S: Studies: Randomized clinical trials and prospective cohort studies.

### 2.2 Definition of Terms

#### 2.2.1 Micro‐Veneered Restorations

Veneering material applied only in the non‐functional buccal areas. Inclusion was based solely on the design description provided in the respective studies. Veneering thickness was not used as a categorization criterion.

#### 2.2.2 Restoration Success

Restorations being present and functional at the time of assessment, without showing any complications and were neither modified nor repaired after a minimum observation period of 1 year.

#### 2.2.3 Restoration Survival

Restorations being present and functional at the time of assessment (after at least one‐year follow‐up), even if complications, modifications, or repair occurred during the follow‐up period.

#### 2.2.4 Complication

Any biological or technical complication occurred after insertion of the restoration. However, not all complications were included in this review (see below).

#### 2.2.5 Chipping

Partial fracture of the ceramic. In the current review, all types of chipping were rated as an event, even if the chipping was minor and polishable.

### 2.3 Search Strategy and Eligibility Criteria

#### 2.3.1 Search Strategy

Based on the PICOS criteria, a search strategy was developed and executed using an electronic search. A systematic electronic search of PubMed MEDLINE, EMBASE, Cochrane Central Register of Controlled Trials (CENTRAL), including the gray literature of Google Scholar was performed for clinical studies up to June 2023. There was no limitation in publication date and no language restrictions. An additional manual search was performed, identifying relevant studies by screening the reference list of all obtained full‐text articles.

#### 2.3.2 Search Protocol

Search pattern was categorized in population, intervention, comparison, and outcome. Each category was assembled from a combination of Medical Subject Headings (MeSH terms) and free‐text words in simple or multiple conjunctions (Table 1).

**TABLE 1:** A list with the keywords (free‐text and MeSH terms) used for the search.

Category	MeSh terms	Free‐text words
Population	Dental implant OR dental implant OR dental implants	Implant OR implants AND dental AND edentulous
	Dental Prosthesis OR dental prosthesis OR partial AND denture AND fixed	Implant‐supported, superstructure OR fixed AND reconstruction OR restoration OR fixed dental prosthesis OR fixed partial denture OR bridge OR FDP OR fixed dental prosth* OR Fixed partial dent*
Intervention	Ceramics	Allceramics OR allceramic OR full‐ceramic OR framework OR veneered framework AND monolithic AND microveneered AND zirconia AND lithium disilicate AND alumina
Comparison	—	Veneered
Outcome	Survival OR failure	Success OR outcome OR complication AND technical AND biological

For the systematic literature search according to the above‐mentioned PICOS criteria, the following inclusion criteria were applied:

- Human clinical studies including randomized clinical trials and prospective studies.
- Studies with at least 10 included participants.
- A follow‐up time of at least 1 year after insertion of restoration.
- Detailed information on the restoration material used.
- Restoration type is clearly described and data from FDPs are reported separately from other types of included restorations.
- In case of multiple publications on the same patient cohort, only the publication with the longest follow‐up time was included.
- FDPs made of all‐ceramic materials, monolithic or veneered, namely zirconia and alumina.
- All brands, kinds of dental implants.
- Sufficient reporting on the detailed clinical outcomes (success, survival, complications) of FDPs.

The exclusion criteria included:

- Not meeting all inclusion criteria
- Retrospective studies, retrospective case series, technical reports, and case reports
- Studies that pool outcomes of different restoration types and materials.
- Studies reporting on metal‐ceramic, metal‐resin, and polyether ether ketone (PEEK) implant restorations.
- Studies reporting on implant‐supported splinted crowns.
- Poor reporting on drop‐outs and number of patients at follow‐up.

### 2.4 Selection of Studies

Two independent reviewers performed the screening process. The search results from all databases were imported to each reviewer's management software. Titles and abstracts were retrieved and assessed for eligibility according to the defined outcomes. For all remaining abstracts, full texts were obtained and independently screened for inclusion. If any titles and abstracts did not provide sufficient information, the full texts were obtained as well. The final selection was based on the above‐mentioned inclusion and exclusion criteria. Again, any disagreement in this stage was discussed between the authors to reach a consensus.

### 2.5 Data Extraction

Data were extracted independently by two reviewers using a data extraction form and tabulated for further analysis. Disagreement was resolved by discussion. The information collected included: author(s), date of publication, study design and setting, follow‐up period, sample size (number of patients/implants/restorations), type of reconstruction (number of units), jaw localization (maxilla/mandible, anterior/posterior), opposing dentition, implant system, restoration design (veneered, micro‐veneered, monolithic), restoration material, retention type (screwed/cemented), manufacturing method, success rate, survival rate, and complications. A detailed description of the obtained data is listed in Table 2.

**TABLE 2:** List of included studies.

Study ID	Study design	Study setting	Mean follow‐up period	No. of patients/dropouts	Number of restorations/dropouts	Type of restoration (no. of units)	Jaw location	Type of implant (bone/tissue level)	Implant material	Brand
Included studies investigating 3–4‐unit FDPs										
Cheng 2018	Prospective	University	24 months	12/0	12/0	3‐unit FDP	Posterior	Bone‐ and tissue‐level	Titanium	Standard and Standard Plus, Straumann, Nobel Biocare
Degidi 2018	Prospective	Private clinic	60 months	76/5	76/1	3‐unit FDP	Posterior	Bone‐level	Titanium	Ankylos, Dentsply
Degidi 2021	Prospective	Private clinic	24 months	50/2	50/1	3‐unit FDP	Posterior	Bone‐level	Titanium	Ankylos, Dentsply
Derksen 2021	RCT	University	12 months	24/0	24/0	3‐unit FDP	Posterior	Tissue‐level	Titanium	Straumann
Pol 2020	Prospective	University	12 months	55/1	59/2	3‐unit FDP	Posterior	Bone‐level	T3 tapered	Zimmer Biomet Dental
Spies 2018	Prospective	University	60 months	13/0	13/0	3‐unit FDP	Posterior	Tissue‐level	Alumina‐reinforced zirconia	Ziraldent FR1, Metoxit AG
Esquivel‐Upshaw 2020	RCT	University	60 months	NR	65/1	3‐unit FDP	Posterior	Bone‐level	Titanium	AstraTech, Dentsply
Ferrini 2018	Prospective	University	36 months	24/0	24/0	3‐ and 4‐unit FDP	Posterior	Bone‐level	NR	NR
Koenig 2019	Prospective	University	24 months	NR/0	12/0	3‐unit FDP	Posterior	Bone‐level	Titanium	Standard and NobelReplace, Straumann
Included studies investigating full arch restorations										
Limmer 2014	Prospective	University	12 months	17/NR	17/0	Full‐arch	Full‐arch	Bone‐level	Titanium	AstraTech, Dentsply
Papaspyridakos 2013	Prospective	University	36 months	14/NR	16/0	Full‐arch	Full‐arch	NR	NR	NR
Cerames 2019	Prospective	Private clinic	20 ± 5.7 months	83/6 (v)		Full‐arch	Full‐arch	Bone‐level	Titanium	Zimmer Biomet
			18 ± 6.5 months	110/10 (m)		Full‐arch	Full‐arch	Bone‐level		

Study ID	Abutment material	Abutment brand	Type of restoration	Restoration material	Type of ceramic	Restoration brand	Veneering material	Veneering brand	Manufacturing method (restoration)	Type of retention	Opposing dentition
Included studies investigating 3–4 unit FDPs											
Cheng 2018	Screw‐retained stock abutments	Straumann or Nobel Biocare	Micro‐veneered	Ceramill Zr/Ceramill Zolid	3Y‐TZP zirconia	Amann Girbach	NR	NR	CAD/CAM	Screwed/cemented	Natural dentition or tooth/implant supported FDPs
Degidi 2018	Screw‐retained standard abutments	Dentsply	Monolithic	Prettau Zirconia	3Y‐TZP zirconia	Zirkonzahn	/	/	CAD/CAM	Tube‐in‐tube	Mostly fixed restorations (2.8% dentures)
Degidi 2021	Screw‐retained standard abutments	Dentsply	Monolithic	Celtra Press	Hot‐pressed Zr reinforced Lithium disilicate	Dentsply Sirona	/	/	Conventional	Tube‐in‐tube	Mostly fixed restorations
Derksen 2021	Ti‐base (non‐engaging)	Straumann	Monolithic	Lava Plus	3Y‐TZP zirconia	3M Espe	/	/	Gr A: conventional impression with scan of mastercast; Gr B: digital with printed model	Screwed	NR
Pol 2020	Individually milled titanium abutments	Zimmer Biomet Dental	Monolithic	LavaPlus High translucency	3Y‐TZP zirconia	3M Espe	/	/	CAD/CAM	Cemented	NR
Spies 2018	Alumina‐reinforced Zirconia (one‐piece implants)	Ziraldent FR1, Metoxit AG	Veneered	IPS emax ZirCAD veneered with IPS emax ZirPress	3Y‐TZP zirconia	Ivoclar Vivadent	IPS emax zirpress	Ivoclar Vivadent	Hybrid (conventional impression, designed with cerecinlab then fabricated manually)	Cemented	Natural dentition, tooth/implant‐supported SCs, RDP
Esquivel‐Upshaw 2020	Gold‐shaded titanium abutments	Atlantis	Veneered	ZirCAD	3Y‐TZP zirconia	Ivoclar vivadent	ZirPress	Ivoclar Vivadent	Hybrid (convetional impression, digitization of model, CAD/CAM manufacturing)	Cemented	Natural dentition
Ferrini 2018	Titanium	17° and 30° extreme abutments, Winsix Biosafin	Veneered	NR	3Y‐TZP zirconia	Nacera Shell	Initial ceramics	GC, USA		Screwed	NR
Koenig 2019	Titanium abutments	1000er Serie‐ Medntika	Monolithic	Lava Plus High translucency	3Y‐TZP zirconia	3 M Espe	/	/	Conventional silicone impression	Screwed except 1 FDP cemented	Tooth/implant FDP
Included studies investigating full arch restorations											
Limmer 2014	20 degree UniAbutments	NR	Monolithic	NR	3Y‐TZP zirconia	Zirkonzahn	/	/	Hybrid (conventional impression, CADCAM fabrication of zirconia restorations)	Screwed	Conventional full‐acrylic denture
Papaspyridakos 2013	NR	NR	Veneered	Procera	3Y‐TZP zirconia	Nobel Biocare	Feldspathic porcelain	NR	Hybrid (conventional impression, cutback 2 mm, scanned then milled zirconia)	Screwed	Implant‐sipported FDPs/RDPs, complete dentures
Cerames 2019	Screw‐retained conical abutments	Zimmer Biomet	Veneered	Prettau zirconia	3Y‐TZP zirconia	Zirkonzahn	ICE Zirkon Ceramics	Zirkonzahn	Hybrid (convetional impression, digitization of model, CAD/CAM manufacturing)	Screwed	NR
			Micro‐veneered	Prettau Zirconia	3Y‐TZP zirconia	Zirkonzahn	NR	NR			

Abbreviation: NR, not reported.

### 2.6 Risk of Bias Assessment of the Included Studies

Assessment of risk of bias in individual studies was made including random sequence generation, allocation concealment, blinding, completeness of outcome data, and selective reporting using the Cochrane Collaboration tool for randomized controlled trials (RoB). The ROBINS‐I tool was used for assessing risk of bias in non‐randomized studies. Here, the quality of studies was assessed according to bias in confounding, participant selection, classification of interventions, measurement of outcomes, and selection of reported results. The final judgment of the risk of included studies according to the ROBINS‐I tool can be “low,” “moderate,” or “high.”

### 2.7 Certainty of Evidence

The critical assessment of the evidence was carried out according to GRADE (Grading of Recommendations, Assessment, Development and Evaluation) [31]. The GRADE system is based on several points such as risk of bias, imprecision, inconsistency, indirectness, and publication bias. It expresses confidence in the body of evidence regarding a specific outcome (survival, complication, and chipping), not to individual studies. The quality of evidence was rated for each outcome in the following categories: very low, low, moderate, and high.

### 2.8 Statistical Analysis

Survival, failure‐complication, and chipping rates were calculated for each study by dividing the number of events by the total exposure time in situ of the i‐FDPs. In most cases, the number of events was extracted directly from the publications. However, the total observation time of the FDPs had to be calculated and included:

1. The observation time of implant‐supported FDPs. That could be followed during the entire follow‐up period.
2. The observation time of an implant‐supported restoration that was lost due to failure during the follow‐up period.

In order to standardize the data, the event rate for each study and outcome was calculated using the following formula:


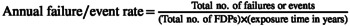
Example: 1 restoration fracture in 12 restorations that were followed up for 2 years: 1/(12 * 2) = 0.04 = 4% annual failure rate (in this study).

For further analysis, Poisson regression models were used, assuming a Poisson distribution of event rates for a given sum of restoration observation years. The Poisson regression models used a logarithmic link function with the total observation time per study as the balancing variable [32]. To reduce the effect of heterogeneity, robust standard errors were calculated to obtain the 95% confidence interval (CI) of the summary estimates of event rates.

To assess heterogeneity in study‐specific event rates, Spearman's rank correlation coefficient and associated *p*‐values were calculated. If the *p*‐value was less than 0.05, indicating heterogeneity, a random‐effects Poisson regression was used to obtain a summary estimate of event rates. One‐ and three‐year survival rates were calculated using the relationship between event rates and the survival function S, where *S* (*T*) = exp. (−*T* * event rate), assuming constant event rates [32]. The 95% CI for survival rates was calculated using the 95% confidence limits of the event rates.

For the comparison of chipping between monolithic and veneered, exact Poisson regression was calculated. To analyze implant loss, the total number of loaded implants served as the denominator. All statistical analyses were performed using STATA (STATA Corporation, College Station, TX, USA), version 15.1.

## 3 Results

### 3.1 Included Studies

**FIGURE 1:**
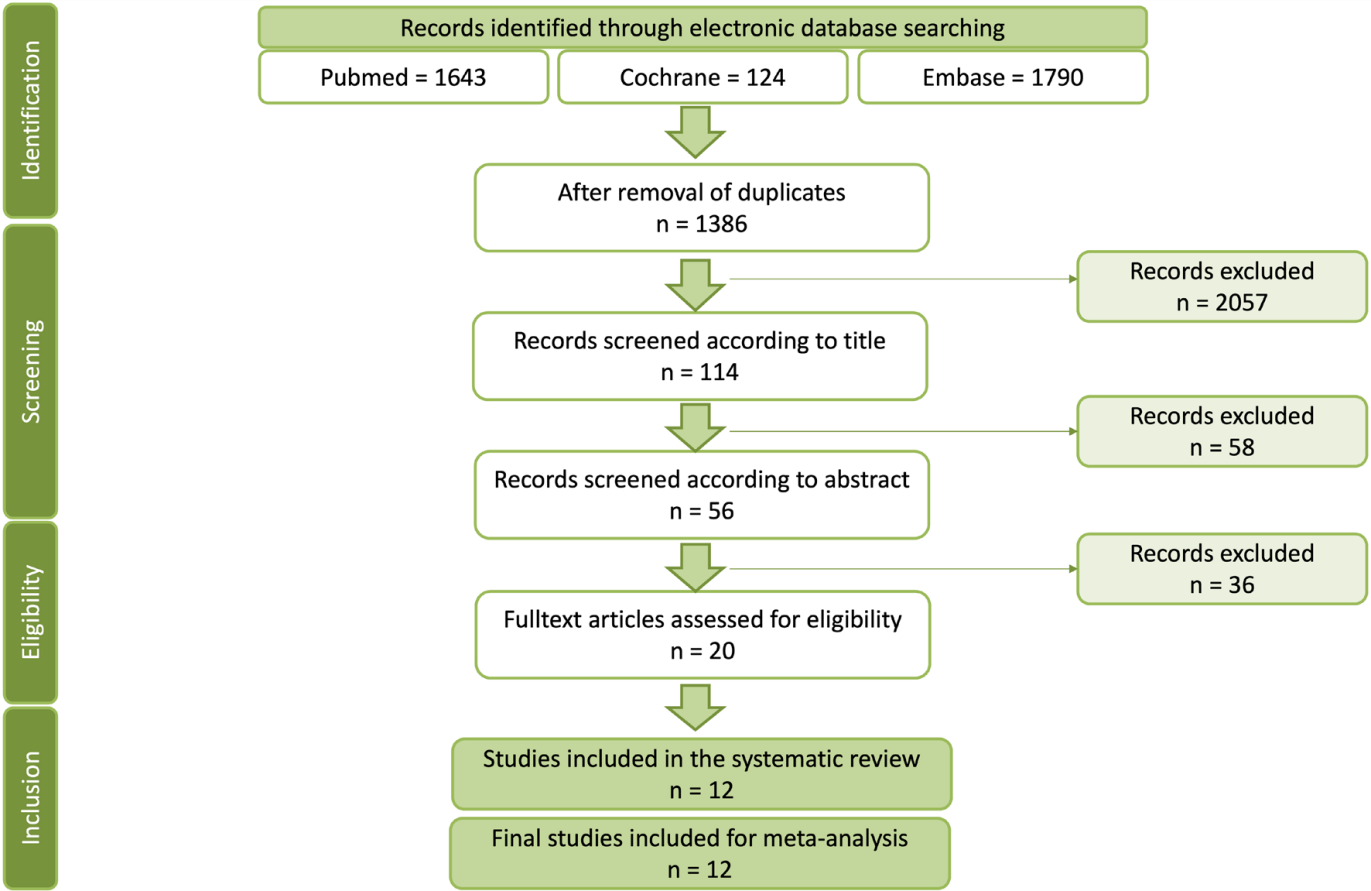
Flowchart of the systematic search results.

The electronic search identified a total of 3577 records. After duplicate removal, a total of 1386 records remained and sorted out by title. The remaining 114 studies were screened according to their abstracts, and finally, 56 studies were selected for full‐text analysis. Twelve studies met the inclusion and exclusion criteria and were included for data extraction. Figure 1 represents the flowchart and selection process.

Publication date of the included studies ranged between 2012 and 2021. Study types were categorized in RCTs (*n* = 2) and prospective cohort studies (*n* = 10). Of the 12 selected studies, 8 studies reported on the outcome of monolithic all‐ceramic i‐FDPs (2 included micro‐veneered restorations) and 4 studies on veneered all‐ceramic i‐FDPs. However, only one of the included studies addressed the focused question exactly in a randomized controlled trial.

### 3.2 Descriptive Analysis

Of the 12 included studies, only one study made a direct comparison related to the aim of the present systematic review comparing monolithic i‐FDPs to veneered ones. Nine of the included studies reported on 3–4 unit i‐FDPs in the posterior region, and three studies investigated implant‐supported full arch restorations. No study reported on anterior i‐FDPs or spans between 5 and 12 restored units. Therefore, the results were reported and analyzed separately. Eight studies reported on monolithic i‐FDPs (2 included micro‐veneered restorations), and four reported on veneered i‐FDPs. Six of the studies reported on screw‐retained implant restorations, three on cemented ones, two studies reported on implant restorations with conometric retention, and one study had a combination of cemented and screwed implant restorations. Except for one study reporting on monolithic hot‐pressed zirconia reinforced lithium disilicate, all included studies reported on tooth‐colored 3Y‐TZP zirconia restorations, either being fully veneered, microveneered, or monolithic. For the overall included restorations, 233 restorations were monolithic, 118 were micro‐veneered, and 181 were veneered implant‐supported restorations.

**FIGURE 2:**
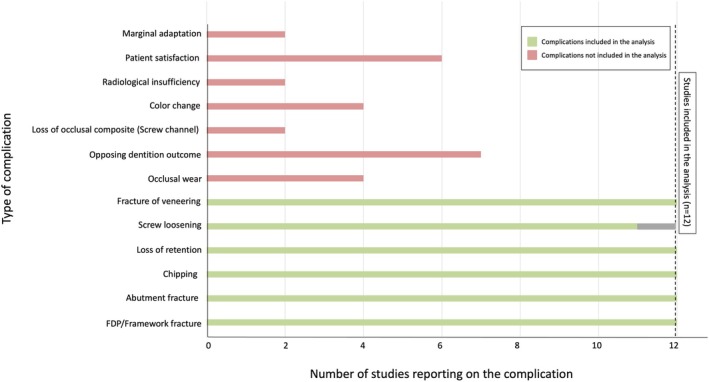
Number of studies reporting on different complications. Only complications documented in the majority of studies were included in the statistical analysis to ensure comparability and reliability. The gray bar related to the complication (screw loosening) indicates one study evaluating cemented implant restorations without the possibility of screw loosening as a complication.

In order to standardize the heterogeneously reported data and create a comparable complication‐free rate only complications that were reported in all studies were selected. These include: (framework) fracture, chipping, screw loosening, loss of retention and abutment fracture. Complications or events assessed as complications such as: anatomic form, color, radiological insufficiency, abrasion, microscopic abrasion analysis, loss of the occlusal screw access channel filling and outcomes of the opposing dentition were not taken into account, because of a lack of uniform reporting. A success‐rate could not be extracted for each study, so that complication‐ and chipping‐rates were defined and evaluated. The composited complication‐free rate was transferred into a success‐rate. The number of studies reporting on each complication is illustrated in Figure 2.

Since the available literature focused primarily on two types of multi‐unit i‐FDPs—3–4 unit i‐FDPs and full‐arch restorations—first, a combined meta‐analysis was conducted where applicable, followed by subgroup analyses specific to each restoration type:

1. Survival, complication, and chipping rate of 3–4 unit i‐FDPs.
2. Survival, complication, and chipping rate of full arch i‐FDPs.

Analyzing all complications from the included studies across all types of restorations (short‐span and full‐arch i‐FDPs) reported that the incidence rate ratio (IRR)—a measure to statistically compare the probability rate of events between groups—was significantly higher for micro‐veneered (IRR = 2.3, *p* = 0.022) and veneered restorations (IRR = 2.8, *p* < 0.001) in comparison to monolithic ones (IRR = 1). When focusing specifically on chipping, micro‐veneered and fully veneered restorations again showed significantly higher incidence rate ratios compared to monolithic designs, in which no incidence of chipping was reported in any of the studies reporting on monolithic restorations.

Regardless of the veneering design, the meta‐analysis reported a statistically significant difference between 3 and 4‐unit FDPs and full‐arch restorations (*p* < 0.001). Conversely, retention type did not significantly affect complication or failure rates (*p* = 0.684), with screw‐retained restorations showing an IRR of 1.15 for complications relative to cement‐retained restorations (IRR = 1.0).

Evaluating the influence of veneering design on the clinical outcome of fixed implant‐supported FDPs was restricted to short span bridges only (3–4 units) due to limited data reporting on full‐arch restorations. For 3–4 unit i‐FDPs, a statistically significant difference between monolithic and veneered restorations in terms of complication and failure rate was reported (*p* < 0.05).

The success, survival, and complication rates of the restorations reported in each included study are summarized in Table 3.

**TABLE 3:** Success, survival and complication rates of the restorations within each included study.

Study ID		Reported FDPs success rate	FDPs survival rate	Annual failure rate	Complications					Calculated annual complication rate	Calculated annual chipping rate
					Abutment fracture	Loss of retention	Screw loosening	FDP fracture	FDP chipping		
Included studies investigating 3–4 unit FDPs											
Cheng 2018		83.3%	91.7%	4%	0	1 (cemented)	0	1	0	4%	0%
Degidi 2018		88.2%	97.4%	0.3%	/	1	0	1	0	2%	0%
Degidi 2021		/	98%	1%	/	0	0	1	0	1%	0%
Derksen 2021		/	100%	0%	/	4	0	0	0	16%	0%
Pol 2020		80.4%	100%	0%	/	3 (not specified whether decementation or screw‐loosening)		0	0	5%	0%
Spies 2018		38.5%	100%	0%	0	0	0	0	7	11%	5%
Esquivel‐Upshaw 2020		78%	96.8%	/	/	NR	NR	NR	16	/	5%
Ferrini 2018		/	100%	0%	0	0	6	0	0	8%	0%
Koenig 2019		100%	100%	/	/	NR	NR	NR	NR	/	0%
Included studies investigating full arch restorations											
Limmer 2014		/	88%	12%	2	1	/	1	0	70%	0%
Papaspyridakos 2013		/	100%	0%	0	0	0	/	5	8%	8%
Cerames 2019	V	87.1%	98.7%	0.6%	/	/	/	1	9	6.5%	6%
	M	90%	99%	0.5%	/	/	/	1	9	5%	4.5%

Abbreviation: NR, not reported.

### 3.3 Three‐ and Four‐Unit Implant Supported FDPs

Nine studies including 330 implant‐supported 3–4‐unit FDPs in the posterior region with a mean follow‐up period of 2.8 years provided data on the survival, chipping, and overall complications of the investigated i‐FDPs. Five studies reporting on 217 restorations with a mean follow‐up time of 2.2 years provided data on the survival of monolithic implant‐supported FDPs [33, 34, 35, 36, 37]. Three studies on 101 restorations after a mean observation period of 4.3 years provided data on the survival and complications of veneered implant‐supported FDPs [38, 39, 40], whereas one study with 12 restorations and a mean follow up period of 2 years reported on micro‐veneered implant‐supported FDPs [41]. No study that fitted the inclusion criteria reported on full‐ceramic cantilever i‐FDPs.

**FIGURE 3:**
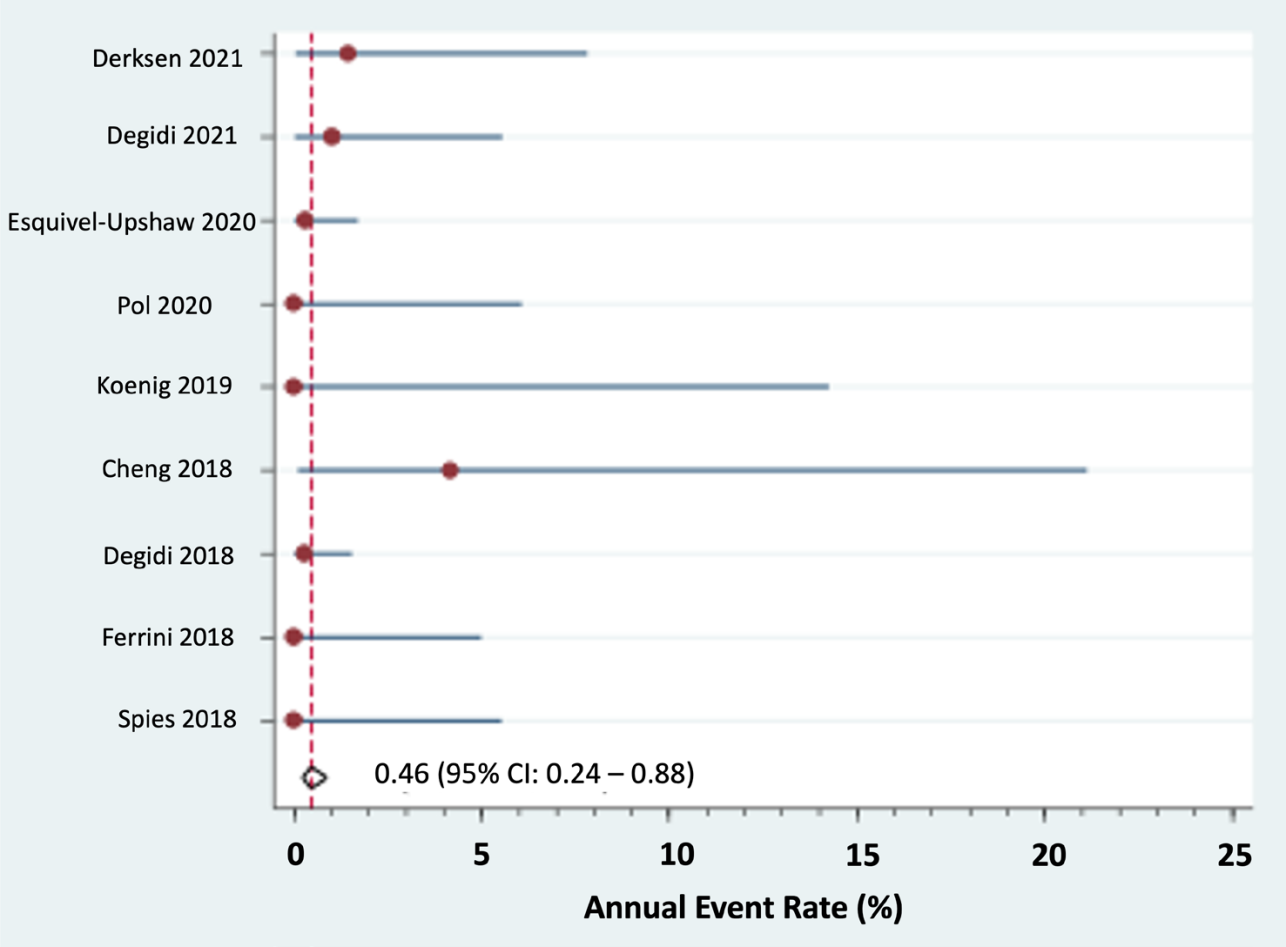
Annual event rate of failure of studies investigating 3–4‐unit implant‐supported FDPs.

The meta‐analysis revealed an estimated overall annual failure rate of all‐ceramic implant‐supported 3–4‐unit FDPs of 0.46% (95% CI: 0.24–0.88), calculated into a 3‐year survival rate of 98.64% (95% CI: 97.40–99.29) (Table 4 and Figure 3).

**TABLE 4:** Calculated survival, complication and chipping rate of 3–4‐unit i‐FDPs using the Poisson regression model.

3–4 unit implant supported FDPs		
Survival	Annual failure rate	0.46% (95% CI: 0.24–0.88)
	3‐year survival rate	98.64% (95% CI: 97.40–99.29)
Chipping	Annual chipping rate	2.15% (95% CI: 0.62–7.37)
	3‐year chipping‐free rate	93.77% (95% CI: 80.17–98.14)
Complications	Annual complication rate	3.78% (95% CI: 1.31–10.70)
	3‐year complication‐free rate	89.36% (95% CI: 72.55–96.14)

**FIGURE 4:**
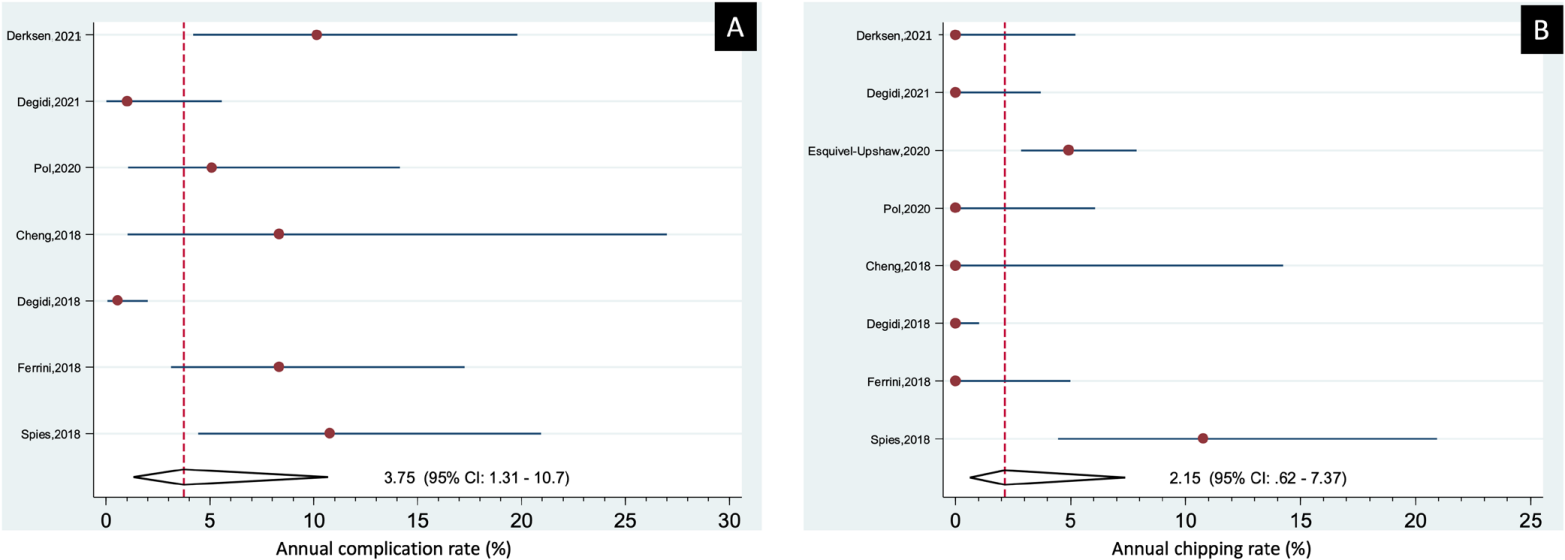
(A) Annual complication rate and (B) annual chipping rate of studies investigating 3–4‐unit implant‐supported FDPs.

Investigating the implant‐supported 3–4‐unit FDPs that failed due to framework fracture showed that two studies with a conometric type of retention reported one 3‐unit FDP failure each after 21 and 41 months [33, 34]. Another study with a cemented implant‐supported 3‐unit FDP also reported one failure due to framework fracture 1 week after cementation [41]. Most of the evidence is based on 3Y‐TZP zirconia‐frameworks or monolithic restorations with a flexural strength of > 1000 MPa However, one study used a monolithic zirconia‐reinforced lithium silicate (ZLS) material, for which the exact flexural strength was not reported [34]. The meta‐analysis revealed an estimated annual complication rate of 3.75% (95% CI: 1.31–10.7), and an estimated annual chipping rate of 2.15% (95% CI: 0.62–7.37) (Figure 4).

Restorations made from monolithic zirconia (including micro‐veneered) exhibited significantly lower incidence rates for chipping (*p* < 0.0001). Other complications included loss of retention, which was reported in four studies [33, 35, 37, 41], screw loosening was reported in one study [39] and two studies with veneered implant‐supported FDPs reported cases with chipping [38, 40]. The study investigating micro‐veneered i‐FDPs reported no incidents of chipping [41]. No abutment fractures occurred among the nine included studies with 3–4 unit FDPs.

### 3.4 Full Arch Implant Supported FDPs

**FIGURE 5:**
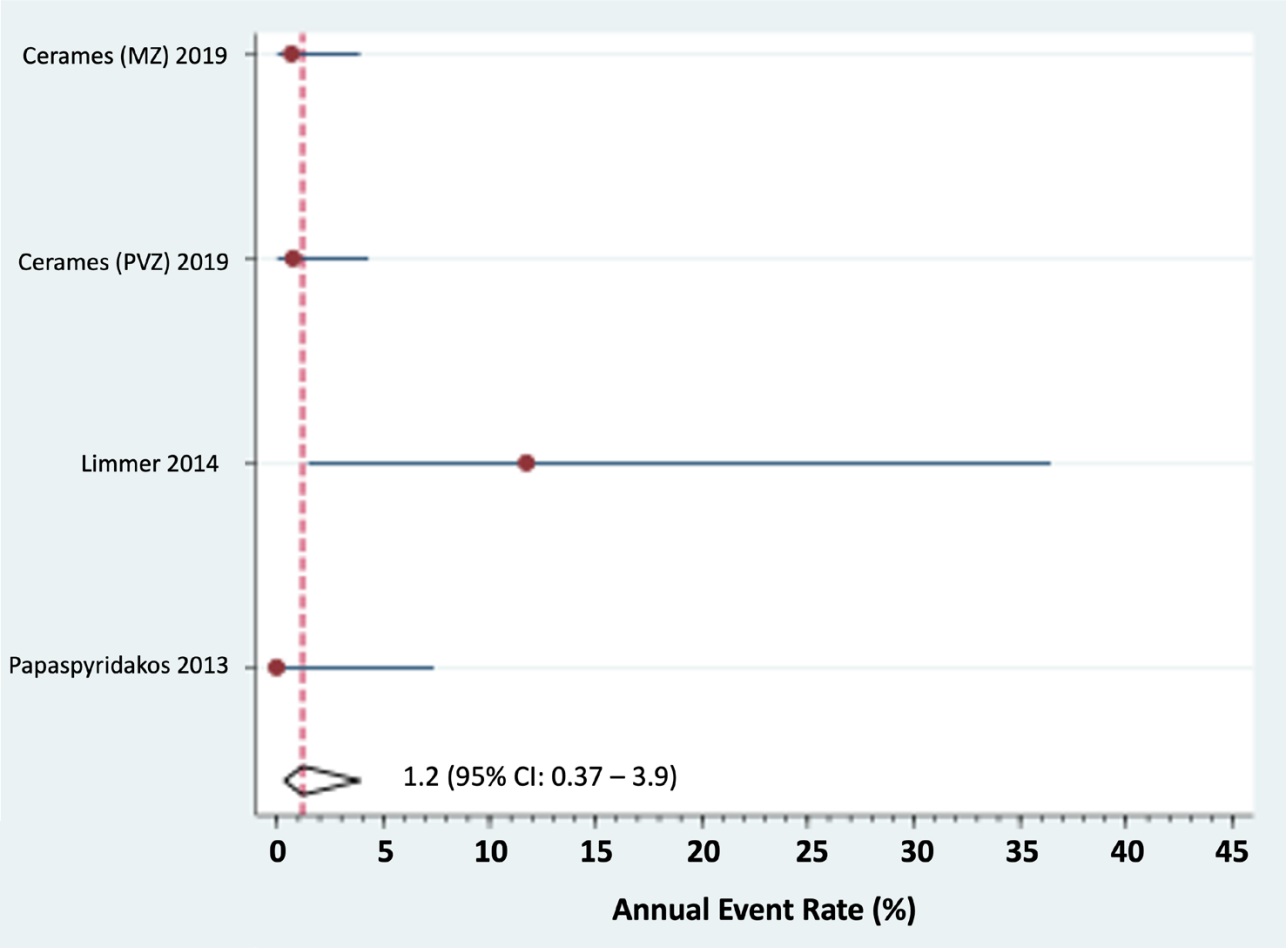
Annual failure rate of studies investigating full‐arch implant‐supported FDPs. MZ, Micro‐veneered zirconia group (Caramês 2019); PVZ, Porcelain‐veneered zirconia group (Caramês 2019).

Three studies including 210 full‐arch implant‐supported FDPs with a mean follow‐up period of 2.4 years provided data on the survival and complications of the investigated restorations. Two studies reporting on 117 restorations after an observation period of 1.2 years provided data on monolithic full‐arch implant‐supported FDPs, and two studies reporting on 93 restorations after a follow‐up time of 2.3 years provided data on veneered i‐FDPs. The meta‐analysis revealed an estimated overall annual failure rate of 1.2% (95% CI: 0.37–3.9), translating into a 3‐year survival rate of 96.45% (95% CI: 88.95–98.88) (Figure 5). However, analyzing the influence of veneering design on the clinical outcome of all‐ceramic full‐arch restorations was not possible due to the limited data available.

**FIGURE 6:**
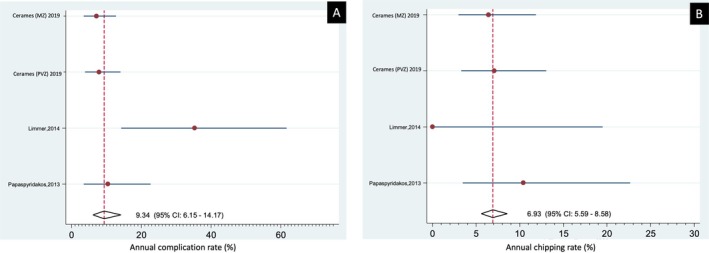
Forest‐plot of (A) annual complication rate and (B) annual chipping rate of studies investigating full‐arch implant‐supported FDPs, MZ, Micro‐veneered zirconia group (Caramês 2019); PVZ, Porcelain‐veneered zirconia group (Caramês 2019).

The evidence is based only on 3Y‐TZP zirconia restorations with a flexural strength of > 1000 MPa. Investigating the full‐arch restorations that failed due to framework fracture revealed that 3 full arch restoration failures occurred in two studies (two monolithic and one veneered) [42, 43]. Other complications included abutment fracture [43], loss of retention [43] and chipping [42, 44]. Chipping was more common in veneered zirconia restorations [42, 44]. A study investigating 17 monolithic zirconia full‐arch restorations reported no chipping; however, other complications were observed, leading to a restoration survival rate of 88%. Most complications affected the opposing maxillary dentures. For the monolithic zirconia full‐arch restorations, recorded complications included fractured abutments (12%), loosened abutments (6%), and one fractured full‐arch restoration [43]. Another prospective clinical study also reported fewer complications in monolithic full‐arch restorations, although chipping was still observed [42]. The study examined 83 implant‐supported, screw‐retained fully veneered zirconia full‐arch restorations and 110 monolithic zirconia full‐arch restorations with buccal veneering. The overall complication rates were 1% for framework fractures in both groups. In the fully veneered group, major ceramic chipping occurred in 5% of cases, and minor chipping in 6.5%, compared to 4% and 5% for major and minor chipping, respectively, in the monolithic group. Despite the overall low incidence of technical complications in both groups, the monolithic restorations demonstrated a lower complication rate than the fully veneered restorations [42]. The meta‐analysis revealed an estimated annual complication rate of 9.34% (95% CI: 6.15–14.17), and an estimated annual chipping rate of 6.93% (95% CI: 5.59–8.58) (Table 5 and Figure 6).

**TABLE 5:** Calculated survival, complication and chipping rate of full‐arch i‐FDPs using the Poisson regression model.

Full‐arch implant supported FDPs		
Survival	Annual failure rate	1.20% (95% CI: 0.37–3.90)
	3‐year survival rate	96.45% (95% CI: 88.96–98.89)
Chipping	Annual chipping rate	6.93% (95% CI: 5.59–8.58)
	3‐year chipping‐free rate	81.23% (95% CI: 77.31–84.55)
Complications	Annual complication rate	9.34% (95% CI: 6.15–14.17)
	3‐year complication‐free rate	75.57 (95% CI: 65.37–83.14)

### 3.5 Risk of Bias Assessment of the Included Studies

**FIGURE 7:**
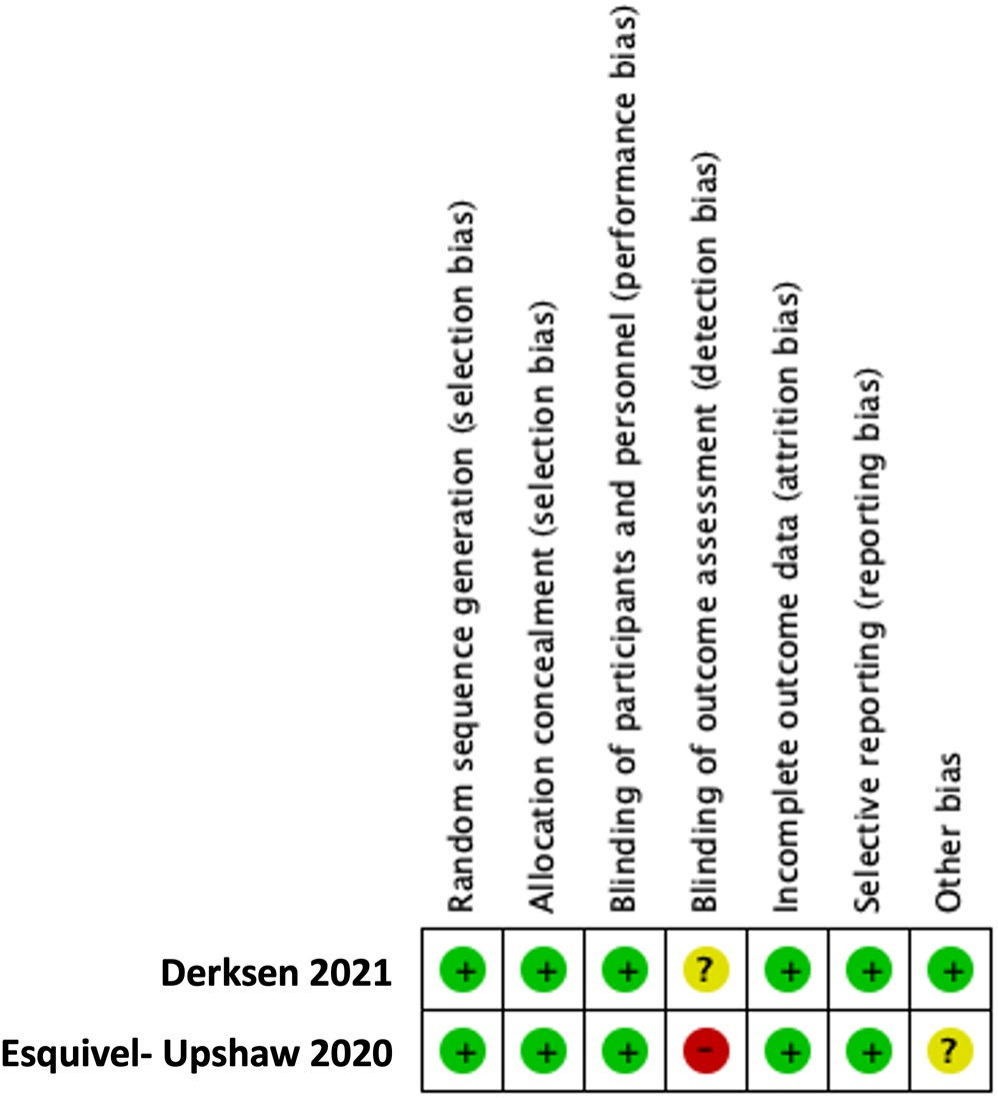
Assessment of risk of bias in the randomized clinical trials using Cochrane Collaboration tool (green: Low risk, yellow: Unclear risk, red: High risk).

Ten of the included studies were prospective and were therefore assessed according to the ROBINS‐I tool (Table 6). Only one study presented an overall low risk of bias [42]. The remaining included studies showed an overall moderate risk of bias. The remaining two studies were RCTs and were assessed using the Cochrane Collaboration tool for randomized controlled trials (RCTs) (Figure 7).

**TABLE 6:** Assessment of risk of bias in the non‐randomized clinical trials using the ROBINS I tool.

Study ID	Bias due to confounding	Bias in selection of participants into the study	Bias in classification of interventions	Bias due to deviations from intended interventions	Bias due to missing data	Bias in measurement of outcomes	Bias in selection of the reported results	Overall bias
Carames 2019	Low risk	Low risk	Low risk	Low risk	Low risk	Low risk	Low risk	Low risk
Cheng 2018	Moderate risk	Low risk	Low risk	Low risk	Low risk	Low risk	Low risk	Moderate risk
Degidi 2018	Moderate risk	Low risk	Low risk	Low risk	Moderate risk	Moderate risk	Moderate risk	Moderate risk
Degidi 2021	Moderate risk	Low risk	Low risk	Low risk	Low risk	Moderate risk	Low risk	Moderate risk
Ferrini 2018	Moderate risk	Moderate risk	Low risk	Low risk	Low risk	Low risk	Low risk	Moderate risk
Koenig 2019	Moderate risk	Low risk	Moderate risk	Moderate risk	Low risk	Moderate risk	Low risk	Moderate risk
Limmer 2014	Moderate risk	Low risk	Low risk	Low risk	Moderate risk	Moderate risk	Low risk	Moderate risk
Papaspyridakos 2013	Low risk	Low risk	Low risk	Low risk	Moderate risk	Moderate risk	Low risk	Moderate risk
Pol 2020	Moderate risk	Low risk	Low risk	Low risk	Moderate risk	Moderate risk	Low risk	Moderate risk
Spies 2018	Moderate risk	Low risk	Moderate risk	Low risk	Low risk	Low risk	Low risk	Moderate risk

## 4 Discussion

The main aim of the current systematic review was to evaluate the survival‐, failure‐, and complication‐rates of monolithic all‐ceramic implant‐supported FDPs in partially and completely edentulous patients in comparison to veneered all‐ceramic i‐FDPs. The meta‐analysis revealed significantly higher complication rates with micro‐veneered and fully veneered restorations in comparison to pure monolithic designs. For 3–4‐unit i‐FDPs, however, the descriptive findings on chipping follow the common clinical and guideline‐based assumption that micro‐veneered restorations behave similarly to monolithic ones. Conversely, for full‐arch restorations, chipping rates were equally high in micro‐veneered and veneered i‐FDPs, which further highlights the increased vulnerability of any veneering design to technical complications. It is important to mention that the available data is limited and none of the included studies directly compared micro‐veneered with monolithic i‐FDPs.

Posterior short‐span implant‐supported FDPs demonstrated high survival rates across all designs over 1–10 years, with an estimated 3‐year survival rate of 98.64%. Ceramic chipping was the dominant technical complication affecting veneered zirconia restorations and reducing overall success. Reported 5‐year chipping rates ranged from 21% to 23% [38, 40], increasing to 69% at 10 years [45]. In contrast, monolithic and micro‐veneered restorations showed consistently lower or absent chipping rates in the included studies [33, 34, 37, 41, 46].

For implant‐supported full‐arch restorations, the current systematic review included three studies, with observation periods of 1–3 years [42, 43, 44]. All three studies reported high survival rates of up to 99%. However, significant complications were observed, including minor and major ceramic chipping with rates of 10%–12% [42] and 31% [44] after 2 and 3 years, respectively, as well as framework and abutment fractures. One larger comparative study including 110 restorations found no significant differences between micro‐veneered and fully veneered designs after 2 years follow‐up period, although micro‐veneered restorations showed slightly lower Kaplan–Meier complication estimates [42]. Limmer et al. showed no chipping in monolithic restorations despite significant complication rates [43], whereas Papaspyridakos et al. showed chipping in 5 out of 16 fully veneered restorations followed up over 2–4 years [44]. Due to the small sample sizes included in the studies, the relatively short follow‐up periods and the low level of evidence, no clear recommendation for the use of monolithic zirconia for full‐arch restorations could be reached.

The fact that osseointegrated implants lack periodontal mechanoreceptors leads to altered neurosensory feedback and potentially higher functional loading. Forces are transmitted directly through the implant–abutment interface, which represents a biomechanically sensitive zone [47, 48, 49]. Occlusal discrepancies have been associated with increased chipping in veneered restorations [50, 51], while their effect on monolithic designs remains unclear [52]. Therefore, material selection is an important factor when restoring edentulous arches with implant‐supported restorations. The prosthetic material and design must be weighed especially when monolithic restorations are planned in order to reduce the risk of technical complications.

Metal‐ceramic restorations were traditionally considered the gold standard for implant‐supported FDPs in high‐load posterior regions. A randomized clinical trial demonstrated comparable performance between veneered zirconia and metal‐ceramic restorations over 5 years, with occlusal forces identified as the primary cause of veneer chipping, while framework and connector design showed no significant effect [38].

With the numerous improvements in composition and microstructure, zirconia is nowadays available in five generations, each with different mechanical and optical properties. In addition, multi‐layer blocks which combine two different generations of zirconia in one block are also available. Here, the generation with good mechanical properties but poorer optical properties is positioned in the area of the bridge framework, whereas the occlusal surface, incisal edge, and labial surface are positioned in the zones with the lower mechanical and increased translucency to ensure better optical properties. These different properties make it clear how important it is for clinicians and dental technicians to be well informed about the different generations and their indications. All studies included in the current systematic review investigated only restorations made from the first [38, 40] and second generation [12, 22, 23, 25, 32]. There are currently no prospective clinical studies with a sufficient observation period for 4‐, 5‐, or 6Y‐TZP (3rd–5th generation) zirconia restorations or multilayers as a combination of several generations in one blank.

The question of whether micro‐veneered restorations can be equated with monolithic designs remains a matter of debate. A major limitation concerns the lack of standardized reporting of micro‐veneering. Although conceptually defined as minimal veneering (≤ 0.5 mm) restricted to non‐functional areas, most studies did not report veneering thickness. Therefore, classification in this review was based on location rather than quantitative measurements, limiting comparability across studies. Additionally, the micro‐veneered subgroup was dominated by one full‐arch study, which may have influenced pooled complication rates [42]. Given that full‐arch restorations inherently show higher complication risks than short‐span FDPs, this imbalance must be considered when interpreting results.

In terms of retention type, screw retention was reported in four of the included studies investigating 3–4‐unit i‐FDPs [35, 36, 39, 41], in which three were screwed to titanium base abutments and in one study multi‐unit abutments were used. In four other studies, the i‐FDPs were either temporarily or adhesively cemented to individual titanium abutments [37, 38, 40, 41]. Two studies used a special screw and cement‐free form of retention known as the cone‐in‐cone connection [33, 34]. Here, the restorations were tapped onto the abutments using a special calibrated tool. The three different types of retention investigated in the included studies showed comparable clinical outcomes across studies. However, complications such as debonding were influenced more by abutment design and bonding protocols than by retention type alone. For full‐arch restorations, all three included studies used screw retained superstructures [42, 43, 44]. Due to the high complication rates mentioned previously, the selection of screw retention is highly recommended when planning full‐arch implant‐supported FDPs to allow predictable retrievability, whenever necessary.

The findings of the current review are consistent with and extend recently published clinical guidelines for all‐ceramic implant‐supported restorations derived from the same body of evidence [53]. In contrast to the limited and predominantly short‐term data available for all‐ceramic full‐arch implant‐supported restorations, metal‐ceramic full‐arch prostheses have been extensively investigated and demonstrate high long‐term survival rates with well‐documented complication profiles. Several long‐term clinical studies report survival rates exceeding 90%–95% at 10 years, with technical complications that are generally manageable and framework fractures being rare [54, 55, 56]. This substantial body of long‐term evidence provides a level of clinical predictability that is currently not yet available for all‐ceramic full‐arch reconstructions.

Consequently, clinicians should prioritize monolithic or micro‐veneered designs for short‐span restorations and carefully weigh the risks of full‐arch all‐ceramic reconstructions, ensuring that patients are adequately informed of potential complications and alternative treatment options.

A strength of this review is the standardized classification of complications, which improved comparability across heterogeneous studies. However, several limitations must be considered, including short follow‐up periods, small sample sizes, and the limited range of restorative materials investigated. Evidence on multilayer zirconia, cantilever designs, and micro‐veneered restorations remains scarce. In addition, functional factors such as occlusal scheme, load distribution, and parafunctional habits—potentially critical for the development of technical complications—are rarely assessed in clinical studies. These limitations highlight the need for well‐designed prospective trials with standardized reporting and longer observation periods.

In summary, monolithic implant‐supported FDPs are associated with lower technical complication rates than veneered and micro‐veneered designs, while maintaining comparable survival rates in short‐span restorations. For 3–4‐unit FDPs, micro‐veneered restorations appear to perform similarly to monolithic designs in the short term. In contrast, full‐arch all‐ceramic restorations demonstrate higher complication rates regardless of veneering approach, and current evidence is insufficient to support a clear preference for monolithic zirconia.

Clinically, monolithic or micro‐veneered designs should be preferred for short‐span restorations, whereas full‐arch all‐ceramic rehabilitations should be planned with caution.

## 5 Conclusions

Within the limitations of this systematic review, it can be concluded that implant‐supported multi‐unit (3–4 units) FDPs in the posterior region and full arch implant‐supported restorations revealed estimated 3‐year survival rates of 98.6% and 96.5%, respectively. For short‐span implant‐supported FDPs, fully monolithic zirconia restorations demonstrated the lowest reported technical complication rates. Micro‐veneered designs may represent an esthetic alternative; however, the available evidence for this design is limited and heterogeneous. Importantly, no clinical studies have evaluated multilayer zirconia. The available data are based exclusively on high strength 3Y‐TZP zirconia with flexural strength exceeding 1000 MPa.

For full‐arch reconstructions, due to the high complication rates reported in the short time frame of 1–3 years and limited data, metal‐ceramic restorations remain the gold standard. Patient information should include these findings when all‐ceramic full‐arch restorations are planned.

## Conflicts of Interest

The authors declare no conflicts of interest.

## Data Availability

The data that support the findings of this study are available from the corresponding author upon reasonable request.
